# Neurochemical Aspects of the Role of Thirst in Body Fluid Homeostasis and Their Significance in Health and Disease: A Literature Review

**DOI:** 10.3390/ijms26167850

**Published:** 2025-08-14

**Authors:** Ewa Szczepanska-Sadowska

**Affiliations:** Laboratory of Centre for Preclinical Research, Department of Experimental and Clinical Physiology, Medical University of Warsaw, 02-097 Warsaw, Poland; eszczepanska@wum.edu.pl

**Keywords:** hypodipsia, polydipsia, renin–angiotensin system, vasopressin, gut peptides, cytokines, gaseous transmitters

## Abstract

Thirst is usually characterized as an unpleasant sensation provoking drinking of water. The purpose of the present review is to draw attention to the importance of thirst in overall regulation of body fluid homeostasis in health and pathology. Intensity of thirst is determined by signals generated in multiple groups of osmosensitive neurons engaged in dipsogenic and antidipsogenic activities, which are located in the brain cortex, the insula, the amygdala, the median preoptic area, the hypothalamic nuclei and the organum vasculosum laminae terminalis. Water ingestion is also influenced by signals generated in the cardiovascular system, the gastrointestinal system, the pancreas, the liver and the kidney and by changes of body temperature. Regulation of thirst engages the autonomic nervous system and several neuroactive factors synthetized in the brain and the peripheral organs. Among them are components of the renin–angiotensin system, vasopressin, atrial natriuretic peptide, cholecystokinin, ghrelin, gaseous transmitters, cytokines and prostaglandins. Experimental studies provide evidence that elevation of fluid osmolality, which is the most frequent cause of thirst, influences function of the voltage-gated sodium channel and calcium-dependent kinase II subunit alpha. Regulation of thirst may be inappropriate in old age and under some pathological conditions including infections, heart failure, diabetes insipidus, diabetes mellitus, and psychogenic disorders. The molecular background of the abnormal regulation of thirst in the clinical disorders is not yet sufficiently recognized and requires further examination.

## 1. Introduction

The physiological and semantic definition of thirst refers to a feeling that is imperative for life. In the present review, the word thirst refers to the need of water ingestion, which is necessary for maintenance of body fluid homeostasis. Humans usually characterize thirst as an unpleasant and distressing experience. Precise regulation of thirst allows one to adjust water intake to the ongoing requirements and permits maintenance of plasma osmolality fluctuations within limits of 1–2%. As hypernatremia is the most essential stimulus of thirst, the intake of water has to be adjusted to salt intake [1,2]. There are two main categories of thirst. Elevated blood osmolality elicited by restricted water intake causes “osmotic thirst”, which is associated with a need of pure water consumption, whereas loss of body fluid, induced by sweating, hypovolemia or gastrointestinal disorders, causes “hypovolemic thirst”, which is associated with an enhanced appetite for electrolytes [3,4]. Thirst can be also modulated by signals generated in the cardiorespiratory and gastrointestinal systems, in the kidney and in the brain regions involved in the regulation of blood pressure, body temperature, stress, emotions and behavior [5,6,7]. Considerable attention has been drawn to the neurochemical background of thirst, particularly to the role of the renin–angiotensin system (RAS), vasopressin and cytokines [5,7,8,9]. Usually thirst can be quenched by consumption of an adequate amount of water; however in the states known as adipsia and polydipsia, water intake may be inappropriate [7,10,11].

Both insufficient drinking and excessive water intake exert negative consequences. In several instances, especially under pathophysiological conditions, multiple signals regulating thirst may act together in complex interdependences and our understanding of these processes is not yet well recognized. The purpose of the present review is to summarize current knowledge on the regulation of thirst and to focus attention on its dysregulation under pathological conditions.

## 2. Methodology

**Figure 1:**
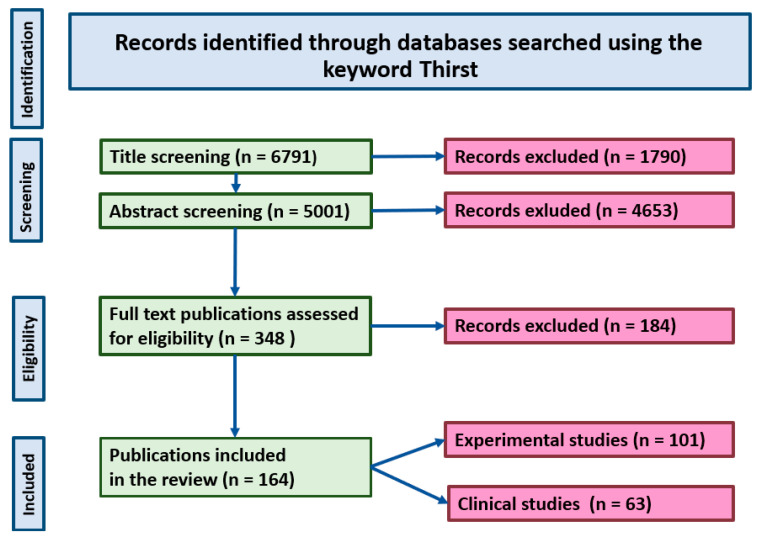
PRISMA diagram showing selection of the studies discussed in the review.

The selection of studies discussed in the present review was performed using the Primary Statement for Preferred Reporting Items For Systematic Reviews and Meta-Analyses (PRISMA) method [12]. The identification of publications through the PRISMA approach was performed through a literature search carried out for studies published between January 1960 and July 2025 with the application of PubMed, EMBASE and Scopus databases. The following criteria were applied for evaluation of each study before its inclusion to the reference list: (1) importance of the study for understanding of the physiology and pathology of thirst, (2) novelty of the study in the specific domain of the thirst science, and (3) the scientific reliability of methods applied in the study, including adequacy of statistical analysis. The PRISMA flow diagram is shown Figure 1. The selection of studies included to the review was based on their relevance to the questions discussed in the review and their scientific contribution.

## 3. Generation of Thirst and Integration of Thirst Signals

### 3.1. Neuroanatomy and Neurochemistry of Thirst

**Figure 2:**
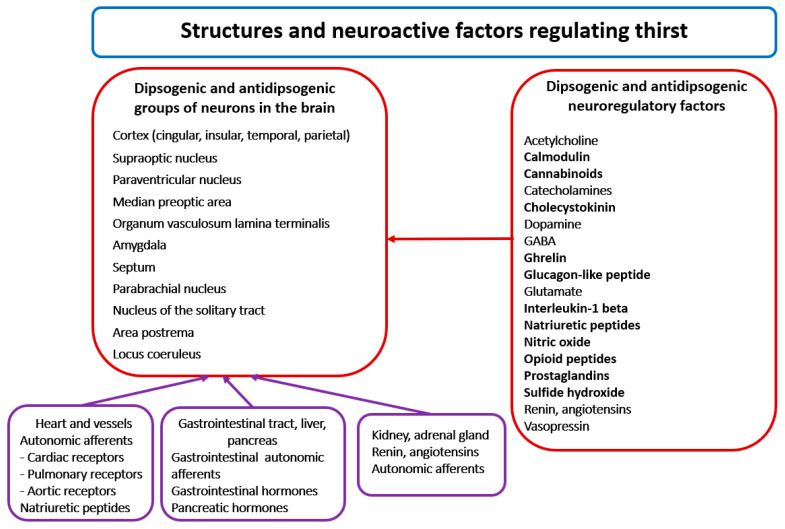
The left part of the figure presents the location of main groups of dipsogenic and antidipsogenic neurons in the brain. The right part of the Figure presents alphabetically arranged list of neuroactive messengers involved in the regulation of thirst. See text for other explanations.

Seventy years ago, Andersson and McCann [13] drew attention to the hypothalamus as the main center of thirst. Later studies allowed the identification specific brain structures involved in initiation and satiation of thirst. At present it is known that the regulation of thirst engages multiple neural networks, forming an extensive thirst system that consists of cooperating groups of neurons located in the median preoptic area (PRA), the paraventricular nucleus (PVN), the amygdala, the septum, the parabrachial nucleus (PBN), the locus coeruleus (LC), the area postrema, the subfornical organ (SFO) and the nucleus of the solitary tract (NTS), as well as in the brain cortex, especially in its anterior cingulate, insular, posterior parietal, middle temporal and superior temporal regions [5,8,14,15,16,17] (Figure 2). It should be noted that neurons of the same regions participate in the regulation of other homeostatic parameters, such as blood pressure, metabolism and body temperature [18]. Lesions of the area postrema and the circumventricular organs (CVOs), such as the SFO and the organum vasculosum laminae terminalis (OVLT), cause deficit of thirst, which indicates that neurons of these regions participate in generation of thirst [19,20].

Regulation of thirst engages multiple excitatory and inhibitory messengers, including all classical neurotransmitters, several neuropeptides, gaseous transmitters and other active molecules [5,14,16,21] (Figure 2).

Early studies on rats provide evidence for involvement of the hypothalamic beta-adrenergic and cholinergic neurons in generation of thirst, and for the essential role of alpha-adrenergic neurons in satiation of thirst. Moreover, it has been shown that an intravenous (iv) administration of the beta-adrenergic agonist isoprenaline stimulates water intake in dogs and that this effect can be prevented by application of the beta-adrenergic receptors antagonist propranolol and intensified by administration of the alpha-antagonist phentolamine [22,23]. More recently it has been shown that the osmotic stimulation of the thirst system is associated with activation of the catecholamine pathways located in the anterior third ventricle area (AV3V), the medial forebrain bundle (MFB) and the supraoptic decussation (SOD). The osmosensitive neurons of the lamina terminalis (LT) project to the insular and cingular cortex and to the basolateral amygdala [24,25]. Lesions of these regions cause adipsia in dehydrated rats [26,27]. Experiments on Long Evans rats provided evidence for a significant role of the mesolimbic dopamine system in evoking thirst during water deprivation and after administration of angiotensin II (Ang II) [27,28]. Studies using the positron emission tomography (PET) in healthy male human subjects showed that thirst induced by intravenous infusion of hypertonic saline is associated with activation of neurons located in the cingulate and the parahippocampal gyri of the cortex, and in the insula, the hypothalamus, the amygdala and the mesencephalon. Moreover, it has been shown that the activation could be abolished by water ingestion [29,30,31]. More recently, application of functional magnetic resonance imaging (MRI) in humans allowed for demonstration that swallowing, which is an inseparable attribute of drinking behavior, is associated with activation of the anterior midcingulate, the premotor and the primary sensorimotor cortex [32].

### 3.2. Primary Role of Osmosensitive Neurons in Regulation of Thirst and Sodium Appetite

The SFO and the OVLT contain excitatory and inhibitory neurons that are activated during osmotic and hypovolemic thirst [3]. Moreover, the osmoreceptive cells of the SFO bear Ang II receptors and project to the vasopressin, producing cells in the supraoptic nucleus (SON) and the paraventricular neurons (PVN) [33,34]. Optogenetic and in vitro studies revealed that in the OVLT, the same neurons can be activated by Ang II and by hypertonic NaCl [34]. In neurons of the SFO and the OVLT, function of sodium sensors is fulfilled by voltage-gated sodium channels (Na_x_, Na(v)2) [35,36,37]. Experiments on male Sprague Dawley rats provided evidence that MPA neurons expressing calcium calmodulin-dependent kinase type II subunit alpha (CaMKIIa) may play a pivotal role in the integration of osmotic thirst with vasopressin release during stimulation by hyperosmolarity and by Ang II [38]. There is evidence for specific engagement of the transit receptor potential cation channel subfamily (TRPV) sensors in the regulation of thirst and in the intrinsic osmosensitivity of OVLT. In addition, it has been shown that TRPV1 channels in the OVLT, the MPA, the SFO and the SON may play essential role in the integration of osmotic thirst and body temperature regulation [39,40,41]. Furthermore, experiments on mice revealed that the intracerebroventricular (ICV) administration of a TRPV4 agonist (4α-PDD) inhibits Ang II-induced water intake, although it does not modify thirst induced by application of hypertonic saline [42].

Osmotic thirst may be also modulated by several other factors. For instance, optogenetic stimulation of the SFO neurons expressing nitric oxide (NO) synthase 1 (NOS-1) revealed that these neurons are activated by administration of hypertonic saline and by Ang II, as well as by hypovolemia elicited by administration of polyethylene glycol and by hypotension induced by administration of isoproterenol [43]. Neurons of the SFO and OVLT manifest prominent intrinsic circadian rhythms that presumably determine their osmosensitivity; however, thus far the neurochemical basis of the control of thirst by biorhythms has not been elucidated [44]. There is evidence that the regulation of thirst by the SFO and the OVLT is coordinated in the basolateral amygdala and in the insula, and that cannabinoid neurons and cannabinoid (CB1) receptors participate in this process [25]. Osmotic thirst activates the cortical regions of the anterior cingulate and the insula, which are known to be involved in the regulation of pain. In this context, it is worth of noting that the perception of thirst is intensified by the pain stimuli [45].

**Figure 3:**
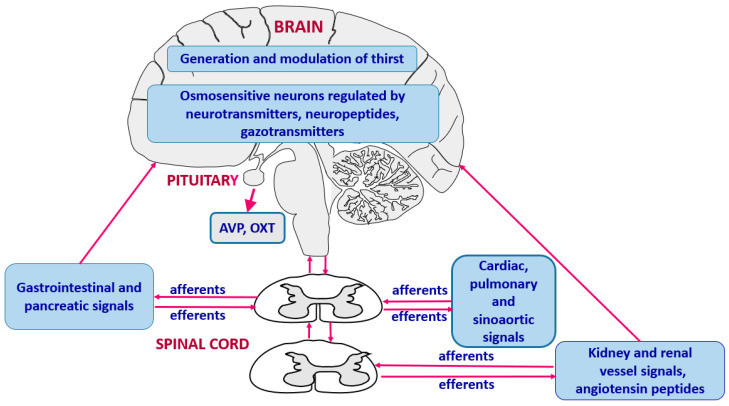
Hierarchical organization and cooperation of central and peripheral neurohumoral mechanisms engaged in generation of thirst. AVP—vasopressin, OXT—oxytocin.

Sodium content gives taste to food and plays an essential role in generation of thirst. There is evidence that the integration of thirst and sodium appetite occurs in the forebrain and circumventricular organs. An especially important role is attributed to the lateral PBN, which receives ascending projections from the NTS [27,46]. In addition, individual excitatory and inhibitory neurons sensing hyperosmotic and hypovolemic thirst are located in the lamina terminalis (LT) [3]. Significant attention has been drawn to Na_x_ channels that belong to a subfamily of voltage-gated sodium channels and are located in astrocytes of the OVLT and SFO [37,47]. Experimental in vitro and in vivo studies indicate that Na_x_ channels play significant role in sensing local sodium concentration by neurons and astrocytes of the CVOs. The operating state of these neurons is modulated by factors regulating thirst and sodium appetite, specifically by cholecystokinin and natriuretic peptides [8,36]. Experimental studies have shown that extracellular dehydration induced by repeated sodium and water depletion may cause distinct sensitization of sodium appetite, which presumably depends on neuroplasticity of glutamate neurons because it can be eliminated by pharmacological blockade of glutamate NMDA receptors [48,49,50]. It is important to note that activity of the brain cells engaged in the regulation of thirst can also be importantly affected by neural and humoral signals generated in peripheral sources, mainly in the heart, vessels, the gastrointestinal system, the pancreas and the kidney. The hierarchical organization of the regulation of thirst is shown in Figure 3, and the cooperation of the central and peripheral factors is discussed in more details in further parts of the review (see Section 3.3, Section 3.4, Section 3.5 and Section 3.6).

### 3.3. Regulation of Thirst by Angiotensin II and Vasopressin

Renin and angiotensin II. Several studies performed on mammalian and submammalian vertebrates show that activation of the RAS plays essential role in the regulation of thirst [2,5,51,52,53]. In dogs and rats, the intracranial administration of Ang II stimulates water intake when the peptide is injected to the lateral and third cerebral ventricles, and to the anterior hypothalamic region, the PRA and the SFO. The most sensitive regions have been located in the PRA and the SFO [51,53,54]. It has been shown that the dipsogenic effect of Ang II is mediated by AT1 receptors [53,55]. Recently, multilateral studies on Sprague Dawley rats using in vitro and in vivo electrophysiology, as well as optogenetic examination of the OVLT neurons, provided evidence that a significant proportion (64%) of the OVLT neurons respond both to hypertonic NaCl and to Ang II [35]. It appears that the RAS starts to be engaged in the regulation of water intake and sodium appetite in a very early period of life, as it has been shown that the offspring rats exposed perinatally to captopril, which is an inhibitor of Ang II formation, drink less water in response to hyperosmotic stimulation, extracellular fluid depletion and β-adrenergic stimulation, and that captopril reduces sodium appetite of the neonates [56].

It is highly possible that angiotensin peptides are engaged in regulation of blood pressure and thirst during cardiovascular disturbances, as it has been shown that the partial obstruction of the aorta above the renal arteries, as well as the reduction of venous return, markedly elevate plasma renin concentration and stimulate water intake in dogs, and that the above effects can be abolished by administration of saralasin, which is a competitive antagonist of Ang II [57,58]. Ang II dependent thirst and sodium appetite can be significantly decreased by intracranial application of atrial natriuretic peptide [59]. Full understanding of the role of particular components of the RAS in the regulation of water intake is hindered by engagement of the RAS in the regulation of blood pressure and fluid balance, which also participate in the regulation of thirst through other mechanisms (see Section 3.6).

Vasopressin. Early experiments on dogs have shown that peripheral administration of vasopressin (AVP, ADH) in subpressor doses decreases the osmotic thirst threshold. In contrast, the pressor doses of AVP reduce the osmotic thirst, presumably through stimulation of the cardiovascular inhibitory input [60]. Subsequent studies have demonstrated that administration of vasopressin V1R and V2R antagonists into the third cerebral ventricle significantly elevate the thirst threshold [61]. Participation of AVP in the regulation of thirst is indirectly supported by studies on patients with the syndrome of inappropriate secretion of ADH (SIADH) and in patients with adipsic hypernatremia. In the SIADH, release of AVP cannot be effectively reduced by hypoosmolality and hyponatremia, and this is associated with lowering of the osmotic thirst threshold. The resetting of osmoregulation in the SIADH syndrome can be corrected by administration of AVP antagonists [62,63,64].

Opposite pathology is observed in patients with adipsic hypernatremia in whom water intake and AVP secretion are not sufficient enough to reduce sodium levels to normonatremic values [65]. Recently, it has been reported that in some of these patients, the adipsic hypernatremia is caused by presence of specific sodium channels (Na_x_) and zinc finger transcription factor (ZSCAN1) antibodies in the pituitary, the hypothalamus and the circumventricular organs [66].

### 3.4. Signals from the Alimentary Tract

Early satiation. Water intake quenches thirst within a few minutes after consumption, even before the fluid is absorbed from the digestive tract, and this is related to rapid activation of several neural circuits [43,67]. In most mammals, water intake strictly matches food consumption and this phenomenon is known as the “prandial drinking”. The process of adjustment of water intake to food intake is not well recognized; however, it is known that food consumption rapidly activates SFO neurons, while water ingestion causes their inhibition [43]. Fluid consumption requires anticipatory identification of water as a safe drinkable fluid. This is associated with activation of specific acid sensing taste receptor cells expressing octopetrin 1 (TRCs) that are present on the tongue [65,68].

Ingestion of water causes inhibition of thirst and sodium appetite via sensory cues, which are activated during distention of the gastrointestinal tract by the ingested fluid [69,70]. Moreover, in dehydrated humans, the temperature of the ingested fluid has an impact on volume of the beverage and the quenching properties of the ingested fluid [71]. Studies on mice showed that generation of thirst and satiation of thirst activate multiple populations of the hypothalamic neurons that project to the other regions of the brain and are engaged in decision-making and goal-directed behavior. It has been shown that optogenetic stimulation of the hypothalamic neurons allows reversal of the dynamics of activation of these neurons during thirst-motivated behavior from thirsty to the pre-satiation state [72].

Gut peptides. Participation of some of the gastrointestinal factors in the regulation of thirst has been well documented. Earlier studies have shown that inhibition of the brain cholecystokinin (CCK) modulates dipsogenic effectiveness of Ang II administered into the PBN [73]. Recent studies revealed that CCK-positive neurons in the SFO are activated by hyponatremia and that they stimulate the gamma-aminobutyric acid (GABAergic) inhibitory neurons. The optogenetic stimulation of neurons expressing CCK mRNA indicated that neurons in the median preoptic nucleus are also engaged in this process [21,74]. Some evidence indicates involvement of glucagon-like peptide-1 (GLP-1) in inhibition of water intake and in enhancement of satiation. For instance blockade of GLP-1 receptors in the brain by exendin was found to potentiate consumption of water provoked by dehydration or by administration of hyperosmotic saline [75]. Furthermore, it has been shown that drinking causes immediate activation of GABAergic neurons of the median preoptic nucleus, which express GLP-1 receptors, and that GABA inhibits thirst neurons in the SFO [67,76]. There is evidence that satiation of osmotic thirst after activation of the GLP-1 receptor pathway engages neurons of the PVN and the NTS [77]. Recently, oral administration of a GLP-1 receptor agonist (semaglutide) has been found to suppress excessive thirst in patients with AVP deficiency [78].

In experiments on ad-libitum-fed and ad-libitum watered rats, ICV administration of obstatin, which is a prohormone of ghrelin, was found to inhibit Ang II-induced water intake [79]. It was also reported that ICV injection of ghrelin reduces the dipsogenic action of ICV administered Ang II and hypertonic NaCl in Sprague Dawley rats, although it does not influence dehydration-induced water consumption [80]. Recently, studies on monkeys have shown that consumption of dry food decreases blood ghrelin concentration; however, no significant correlation was found between blood ghrelin and osmolality levels [4]. In healthy men, dehydration-induced exercise decreased ghrelin concentration and increased neural responses, illustrating thirst intensity [81]. However, it should be noted that the role of ghrelin in the regulation of thirst is not fully clear because iv infusion of acyl ghrelin was found to elicit thirst in human patients with the hypopituitary [82].

### 3.5. Gaseous Transmitters, Cytokines and Prostaglandins

Experimental studies show that brain neurons engaged in the regulation of thirst are able to synthesize gaseous transmitters (nitric oxide, NO; sulfide hydroxide, SH_2_) and that these compounds modulate thirst sensation. Furthermore, it has been shown that ICV administration of L-NAME (Ng-nitro-L-arginine methyl ester), which is an inhibitor of nitric oxide synthase, reduces water intake after administration of hypertonic saline or hemorrhage [83]. In contrast, ICV injection of L-arginine, the precursor of NO, has been shown to reduce water intake induced by dehydration or by ICV administration of Ang II [84,85]. Moreover, 24-h fluid deprivation was found to activate the sulfide generating enzyme in the hypothalamus, whereas ICV administration of an SH_2_ donor (NaS_2_) reduced water intake elicited by water deprivation [86]

It is possible that cytokines exert an antidipsogenic action because the intraperitoneal and/or central administration of interleukin-1 beta (IL-1 beta) has been found to reduce water intake during hypovolemia and after administration of hypertonic saline [87,88]. As the antidipsogenic action of IL-1 beta could be abolished by inhibition of prostaglandin (PG) production and by blockade of the central opioid system it was suggested that PGs and opioids participate in the antidipsogenic activity of this cytokine [87,88].

### 3.6. Cardiovascular Regulatory Signals

Experiments on rats and pigs revealed that isotonic reduction of body fluid induced by administration of furosemide or bleeding increases water intake [89,90]. It has also been shown that hypovolemic thirst is caused by reduced stimulation of the cardiovascular receptors and by activation of the RAS. In support of this statement are the findings that reduced venous return or partial aortic obstruction elevate plasma renin level and increase the intake of water and that both these effects can be reduced by inhibition of the systemic and central RAS [57,58,91]. In contrast, overloading of the heart in dogs with the arteriovenous fistula reduced water intake and water/food ratio [92]. Similarly, distention of the left pulmonary vein at its junction with the left ventricle effectively reduced ingestion of water that was elicited by application of dipsogenic stimuli, such as injection of isoproterenol, infusion of hypertonic NaCl, or overnight water deprivation [93]. Evidence has been provided for the importance of the neurogenic component of the cardiovascular inhibitory responses because blockade of the left vagosympathetic nerve prevented the inhibitory effect of pulmonary vein distension on stimulation of thirst by isoproterenol [93]. Furthermore, it has been shown that denervation of the sinoaortic baroreceptors significantly enhances water intake induced by systemic administration of Ang II or hyperosmotic (1 M) saline [94]. Current evidence indicates that the central components of the RAS produced in the OVLT, the SFO, the PVN, the PBN and the rostral ventrolateral medulla (RVLM) play significant role in the regulation of blood pressure and in the modulation of thirst in cardiovascular disorders [35,52,54,95]. Formation of local cooperating RAS-dependent neuronal circuits that are engaged both in the regulation of blood pressure and body fluid balance may play primary role in body fluid maintenance and cardiovascular homeostasis.

## 4. Other Factors Influencing Thirst

### 4.1. Heat, Fever and Heat Acclimation

Heat exposure may cause thermal dehydration, which is followed by stimulation of thirst. Apart from that, there is a direct impact of body temperature on regulation of water intake. Early studies on dogs showed that local elevation of temperature in the preoptic region of the brain elevates the osmotic thirst threshold in dogs, indicating that the temperature of this region may play a role in inhibition of thirst sensation during hyperthermia [96]. Subsequently, it has been shown that the osmoregulatory regions of the brain possess ΔN-TRPV1 channels, which respond both to temperature and osmolality changes and presumably may participate in co-detection of body temperature and body fluid tonicity [41]. A significant increase of the thirst threshold was found during hyperthermia elicited by administration of an uncoupling agent (2,4-dinitrophenol) and during pyrogen-induced fever [97,98]. Inhibition of thirst associated with increased production of prostaglandins (PGI_2_), nitrates, cytokines and melatonin was observed in experiments on rats receiving *Escherichia coli* lipopolysaccharide treatments. The authors suggested that the lipopolysaccharide induces release of melatonin, which subsequently enhances production of NO and inhibits thirst via the nitric oxide–PGI_2_ pathway [99,100,101,102]. It has been postulated that during the pyrogen fever, the antidipsogenic effect of prostaglandin I_2_ (PGI_2_) is mediated through the action exerted in the preoptic area [99].

Studies on rats revealed that heat acclimation causes suppression of thirst induced by dehydration; however, it enhances the dipsogenic action of Ang II injected into the jugular vein [103]. Other studies revealed that water intake is greater in hot–humid environments than in hot–arid environments and that fluid ingestion is influenced by climate properties, such as environmental temperature and humidity, and can vary cross-culturally [104]. In addition some specific properties of the ingested fluid may play a role in satiation of thirst. For instance, in dehydrated humans, the thirst quenching properties of the ingested beverage can be modulated by coldness of the liquid [71]. On the other hand, the palatability of the fluid does not appear to determine volume of the liquor taken during exercise performed in the heat [105].

### 4.2. Aging

In elderly men, the osmotic thirst threshold is elevated and the total fluid intake is reduced, and this indicates that aging is associated with reduced sensation of thirst [106,107,108]. Studies on Fischer 344/Brown Norway F1 rats and Munich Wistar rats confirm reduced sensitivity of the thirst system to hyperosmotic stimuli in old animals, and show that the thirst system of the old rats is also less sensitive to thermal dehydration, angiotensin and hypovolemia [109,110,111,112]. Furthermore, it has been shown that the administration of captopril augments ingestion of water and saline in young animals, whereas it is ineffective in old individuals [113,114]. Recent experimental studies suggest that suppression of thirst in aging animals is caused by elevated production of prostaglandin E_2_ (PGE_2_) and NO, which results from enhanced activation of PGE synthase and inducible NO synthase (iNOS) in the hypothalamic cells [109]. Thus far, the regulation of thirst in old humans is not sufficiently recognized. Old people frequently suffer from dryness of mouth, which forces moistening of the mouth and interferes with evaluation of other thirst regulating signals. In addition, the old patients may have problems in communication of their needs [115].

## 5. Regulation of Thirst in Pathological Conditions

### 5.1. Heart Failure

In 1975, Ramsay et al. [116] reported that heart failure, produced in dogs by constriction of the inferior vena cava in the thoracic region, significantly increases water intake. They also found that enhancement of thirst can be significantly reduced by administration of saralasine acetate, which is a competitive inhibitor of angiotensin [116]. Similarly, enhanced thirst was observed in dogs with reduced venous return elicited by inflation of a balloon in the abdominal region of the aorta [58]. On the other hand, formation of a fistula between the femoral artery and the vena cava, which is associated with elevation of the central venous pressure, arterial blood pressure and extracellular fluid volume, significantly decreased daily water intake [92]. Subsequently, it has been found that ischemic heart disease, which is the most common cause of the heart failure, stimulates thirst in association with activation of the sympathetic nervous system, the RAS and other cardiovascular regulatory mechanisms [18]. It has been also reported that prolonged activation of these responses results in the progression of heart failure [117,118,119,120].

Clinical observations provide evidence that elevated thirst is a characteristic symptom of heart failure and determines the quality of life [120,121,122,123,124,125]. Restriction of water intake is usually recommended in patients suffering from the heart failure as a method for preventing cardiac overloading. However, in some patients, especially in those prescribed high doses of loop diuretics or receiving inhibitors of Ang II receptors, the exaggerated thirst is not observed, presumably because of abolishment of the stimulatory effect of Ang II on thirst. Thus, the recommendation of fluid restriction requires careful analysis in individual patients and has to be adjusted to the hemodynamic conditions of the patient and the type of treatment applied [126].

### 5.2. Diabetes

Significant loss of water stimulates thirst both in diabetes insipidus and in diabetes mellitus. In most instances water intake compensates water losses; however, in some diabetic patients, abnormalities in the regulation of thirst have been reported.

Central and nephrogenic diabetes insipidus. Increased thirst is an inherent attribute of diabetes insipidus and usually properly compensates excessive water losses that are caused by insufficient secretion or action of vasopressin [127,128,129,130]. Deficient thirst and adipsia were observed in patients with adipsic diabetes insipidus [129,131,132]. Particularly alarming were the clinical conditions of patients in whom adipsia was associated with significant hypernatremia [133,134,135]. Disturbances of thirst are frequently observed in patients with brain trauma. Traumatic lesions may directly affect the brain neurons secreting AVP. They may also cause a disruption of the blood–brain barrier (BBB). Injury of the BBB may result in an inappropriate exchange of compounds engaged in the regulation thirst between the brain and the rest of the body [136,137,138].

Diabetes mellitus. Most frequently, diabetes mellitus is caused by an inappropriate production or action of insulin. According to current classification there are two main types of the diabetes mellitus known as diabetes mellitus of type 1 (juvenile-onset, T1D) and diabetes mellitus of type 2 (adult-onset, T2D), which are divided to several subcategories [139]. Hyperglycemia, which is a typical symptom of diabetes mellitus, causes osmotic diuresis, resulting in hypertonicity and hypovolemia that stimulate thirst. In addition, patients with diabetes mellitus of type 2 frequently overeat and are obese, whereas it has been shown that overeating human subjects also manifest elevated thirst [140].

Usually water intake in patients with diabetes mellitus is adequate to water loss [141,142]; however, in some patients regulation of thirst is not sufficient and hyperglycemia may be associated with hyperosmolality and hypernatremia [143,144]. The reasons for this abnormality are not fully understood. Some studies provide evidence that metabolic disorders disturb the release of AVP and activation of thirst. For instance it has been found that the metabolic disturbance, induced by iv administration of the competitive inhibitor of glucose utilization (2-deoxy-D-glucose) in healthy male volunteers elicits elevation of plasma AVP level and enhances thirst. These effects could not be sufficiently explained by changes of plasma osmolality, stimulation of peripheral adrenergic receptors or increase of plasma renin activity [145]. Recently, it has been emphasized that early detection of diabetes mellitus may markedly help to avoid secondary complications related to inappropriate control of thirst by neuroendocrine factors [146,147].

### 5.3. Psychogenic Thirst Abnormalities

Thirst is regulated by several groups of neurons, which interact with multiple neural circuits involved in the regulation of emotions, mood and behavior. Patients with psychiatric diseases frequently manifest abnormal regulation of thirst, such as compulsive thirst or deficient drinking. The dysregulation of water intake may render difficult medical management of these patients [148]. Psychogenic polydipsia and enhanced AVP secretion associated with hyponatremia were reported in patients with schizophrenia [149,150,151,152,153]. Some evidence indicates that in patients with schizophrenia, the abnormalities of polydipsia, hyponatremia and neuroendocrine regulation are associated with pathology of the anterior hippocampus and the prefrontal/limbic brain regions [154]. In contrast, hypodipsia with concomitant hypernatremia were reported in other patients with mild psychogenic disorders in whom the symptoms have been attributed to deficient secretion of vasopressin [155,156,157]. Disturbances of thirst associated with severe hypernatremia and dehydration were also observed in patients with depression and mood disorders [158]. Although it is not possible to imitate well human psychiatric diseases in animal models, it should be noted that chronic dehydration was found to decrease sensitivity to intracranial self-stimulation of the brain reward regions in Long Evans rats, thereby suggesting that proper hydration plays a role in perception of reward effects and determination of emotions [159].

**Figure 4:**
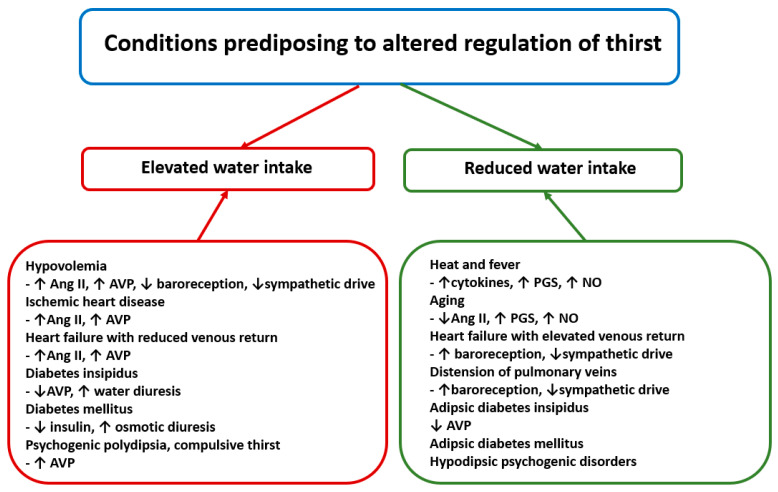
The left part of the figure presents clinical conditions in which daily water intake exceeds average intake. The right part of the figure presents conditions in which daily water intake is lower than average daily water intake. Ang II—angiotensin II, AVP—arginine vasopressin, NO—nitric oxide and PGS—prostaglandins. See text for other explanations.

Conditions predisposing to inappropriate regulation of thirst are summarized in Figure 4.

## 6. Limitations and Future Directions

In surveying the experimental and clinical studies, the complexity of mechanisms responsible for appropriate generation and inhibition of thirst stands out. Growing evidence indicates the importance of complex regulation of thirst by signals generated in the brain, the cardiovascular system, the alimentary tract, the pancreas, the liver and the kidney. However, in spite of significant progress in the knowledge of thirst, there are still several deficits in this field. Former studies concentrated mainly on behavioral symptoms of thirst and did not allow for correlation of these symptoms with biomolecular processes (for instance up-regulation or down-regulation of receptors and the post-receptor events), which may occur in cells engaged in the regulation of thirst. It is likely that better recognition of these processes would allow elucidation of discrepancies in the dipsogenic properties of some bioactive compounds that are observed after their application in different doses or through different routes. It should be also emphasized that the molecular mechanisms responsible for the activation and inhibition of thirst have been investigated in experiments performed mainly on animals and their results should be transferred to humans with an appropriate caution. Furthermore it should be noted that the current knowledge of thirst physiology in animals is based on experiments performed on mammals (rats, mice, dogs, goats, pigs, monkeys) and cannot be referred to other species (for instance birds, bees and fishes). Apparently, the regulation of water intake in these species may be worth of attention in future investigations.

Future studies should more carefully evaluate thirst in human patients suffering from the neuropsychiatric, cardiovascular, visceral, metabolic and renal diseases, in which regulation of thirst may be altered. Evaluation of thirst in humans may be especially difficult in subjects who are not able to communicate their needs for water, such as neonates or unconscious patients [160,161]. The disproportion in the knowledge of thirst in experimental and clinical studies results mainly from ethical restrictions, which are encountered by investigators evaluating thirst in humans with currently available methods. Therefore future studies should focus on elaboration of more efficient biomolecular determinants of body hydration, which would reliably correspond to intensity of thirst.

## 7. Summary

This review summarizes current knowledge on the regulation of thirst in health and in pathological disorders. Sensation of thirst is a consequence of dehydration of osmosensitive neurons due to elevation of body fluid osmolality. The main groups of osmosensitive neurons are present in the brain. The osmosensitive cells have also been found in the liver and the gastrointestinal tract [162,163], and it is possible that signals from these cells may participate in the control of water and/or salt ingestion. Thirst is also modulated by signals generated in cardiovascular receptors and gastrointestinal stretch receptors. It is also regulated by several neuromodulatory factors generated in the brain and peripheral tissues during changes of blood volume and pressure, as well as during food ingestion. In most instances ingestion of water corresponds well to water demands; however, in old age, during infectious diseases associated with fever, in heart failure and in metabolic diseases, as well as in some psychogenic disorders, the regulation of thirst may be abnormal and hyperdipsia or hypodipsia may create clinical problems. The knowledge of complex mechanisms underlying cooperation of neurogenic, humoral and hormonal mechanisms engaged in the regulation of thirst is not yet satisfactory and requires better understanding with the use of a cross-disciplinary holistic approach. In this review, the outstanding contribution of James T Fitzsimons (1928–2023) to the development of thirst physiology is frequently reported [5,9,22,51,52,53,54,55,57,58,90,91,93]. His studies performed in the Physiological Laboratory in Cambridge, and his engagement in the activity of the Commission of Food and Fluid Intake of the International Union of Physiological Sciences greatly contributed to the progress of thirst physiology.

## 8. Conclusions

1. Thirst is activated during elevation of the osmolality of body fluid, which causes dehydration of osmosensitive neurons located in multiple regions of the brain and in some peripheral organs.
2. Proper neurogenic and endocrine regulation of thirst is based on multiple micro- and macro-feedback interdependences that allows maintenance of body fluid osmolality with a precision of 1–2%.
3. Stimulation and inhibition of thirst can be modulated by signals generated in the brain and in the peripheral organs during changes of blood pressure, blood volume, body temperature and digestive processes, especially in situations disturbing homeostatic conditions.
4. Abnormal regulation of thirst that may be associated with inappropriate salt appetite is observed during hyperthermia, during aging, in heart failure, in diabetes insipidus and diabetes mellitus and in some psychogenic disorders.
5. The molecular background of the abnormal regulation of thirst under pathological conditions is not yet well recognized. Complexity of these processes may require application of a sophisticated mathematical approach, which may be necessary to define multiple interactions of cellular processes during homeostatic disorders that challenge thirst regulation.
6. Currently available studies do not allow for correlation of the behavioral symptoms of thirst with molecular processes occurring in cells in the same experimental models. Progress in this field is necessary for elaboration of more effective dipsogenic and antidipsogenic treatments.
7. Regulation of thirst in human beings is not fully recognized. Future studies should more deeply investigate thirst in patients suffering from the neuropsychiatric, cardiovascular, visceral, metabolic and renal diseases, especially when these diseases occur jointly.
8. Future studies should elaborate more efficient biomolecular determinants of body hydration, which would correspond to the intensity of thirst and allow evaluation of thirst in human patients who are not able to communicate their water needs.

## Data Availability

Not applicable.

## Abbreviations

The following abbreviations have been used throughout the text: Ang II—angiotensin II, ADH—antidiuretic hormone, AVP—arginine vasopressin, AV3V—anterior area of the wall of the third ventricle, BBB—blood–brain barrier, CB—cannabinoid, CCK—cholecystokinin, CVO—circumventricular organ, GABA—gamma aminobutyric acid, GLP—glucagon-like peptide, ICV—intracerebroventricular, IL—interleukin, L-NAME—(ng-nitro-L-arginine methyl ester), LC—locus coeruleus, LT—lamina terminalis, MFB—medial forebrain bundle, MPA—median preoptic area, NMDA–N-methyl-D-aspartate receptor, NO—nitric oxide, NOS—nitric oxide synthase, NTS—nucleus of the solitary tract, OVLT—organum vasculosum laminae terminalis, PBA—parabrachial nucleus, PG—prostaglandin, PRA—preoptic area, PRISMA–Primary Statement for Preferred Reporting Items For Systematic Reviews and Meta-Analyses, PVN—paraventricular nucleus, RAS—renin–angiotensin system, RVLM—rostral ventrolateral medulla, SFO—subfornical organ, SH_2_—sulfide hydroxide, SIADH—syndrome of inappropriate secretion of ADH, SON—supraoptic nucleus, T1D—diabetes mellitus of type 1, T2D—diabetes mellitus of type 2, TRPV—transit receptor potential cation channel, and V1R and V2R—vasopressin receptors.
